# Hybrid photosynthesis-powering biocatalysts with solar energy captured by inorganic devices

**DOI:** 10.1186/s13068-017-0943-5

**Published:** 2017-10-30

**Authors:** Tian Zhang, Pier-Luc Tremblay

**Affiliations:** School of Chemistry, Chemical Engineering and Life Science, Wuhan University of Technology, Wuhan, 430070 People’s Republic of China

**Keywords:** Hybrid photosynthesis, Biocatalyst, Photocatalyst, Photobioelectrochemical cell, Microbial electrosynthesis, Photovoltaic, Biofuel, CO_2_ reduction, Solar energy

## Abstract

The biological reduction of CO_2_ driven by sunlight via photosynthesis is a crucial process for life on earth. However, the conversion efficiency of solar energy to biomass by natural photosynthesis is low. This translates in bioproduction processes relying on natural photosynthesis that are inefficient energetically. Recently, hybrid photosynthetic technologies with the potential of significantly increasing the efficiency of solar energy conversion to products have been developed. In these systems, the reduction of CO_2_ into biofuels or other chemicals of interest by biocatalysts is driven by solar energy captured with inorganic devices such as photovoltaic cells or photoelectrodes. Here, we explore hybrid photosynthesis and examine the strategies being deployed to improve this biotechnology.

## Background

Fossil fuels still represent today more than 80% of all the energy sources employed in the world [1]. Besides being used as fuels, petroleum, coal, and natural gas are also major precursors for the industrial production of a large range of chemicals [2]. Fossil fuels are finite resources, and their indiscriminate utilization causes significant harmful effects on the environment, such as oil spill and water pollution. One of the major negative environmental impacts of fossil fuels is the release of large quantity of CO_2_ in the atmosphere representing 65% of all the greenhouse gas emissions responsible for anthropogenic global warming [3].

Because of the critical disadvantages associated with the extensive usage of fossil fuels, the sustainable and economically viable production of fuels and other chemicals from CO_2_ and renewable energy sources has become one of the main technological challenges of our time. Besides being the main driver for climate changes [4], CO_2_ is also a renewable resource and a major carbon source for living organisms. In terms of renewable energy, sunlight is very abundant with ca. 885 million TWh of power reaching the Earth surface every year. According to the International Energy Agency (IEA), this represents 3500 times the total quantity of energy that would be used by human in 2050 [5, 6]. Consequently, it is sensible to undertake research efforts aiming at powering CO_2_ reduction by means of solar energy at industrial scale, which is currently the pursuit of multiple research groups around the world.

Solar energy has two important constraints that must be considered for the elaboration of novel technologies: its intermittent nature and its low average terrestrial power density (global mean of 170 w m^−2^) [7, 8]. Thus, it is of great importance to conceive systems with optimal efficiency for the capture of solar energy and its subsequent conversion in fuels that are easy to store and use.

Over billions of years, evolution has developed natural photosynthesis to convert solar energy into chemical energy to power living cells [9]. Until now, many of the bioproduction processes employing living organisms as catalysts for the synthesis of fuels or other chemicals have relied on natural photosynthesis to acquire the necessary energy. This can be done directly by means of photosynthetic plants, algae, microalgae, or bacteria, converting inorganic carbon molecules into products [10–14]. Alternatively, this can also be done indirectly via the oxidation or fermentation of organic carbon molecules derived from photosynthetic biomass by nonphotosynthetic microorganisms [15–19]. However, natural photosynthesis has evolved over time to facilitate the reproductive success of photosynthetic organisms and not for high efficiency in terms of conversion of solar energy-to-biomass or solar energy-to-specific chemicals [7]. For instance, sugarcane fermentation for the production of ethanol has an average solar energy-to-product conversion efficiency of only 0.2%, whereas the efficiency of photosynthetic production of oil by microalgae is higher but still very low at 1.5% [20–23]. Because of this limited solar energy conversion efficiency, which also has a negative impact on agricultural yields, recent efforts have been deployed to engineer photosynthetic organisms via different synthetic biology strategies to improve solar energy-to-biomass conversion [24–28].

In comparison with natural photosynthesis-based processes, artificial photosynthesis systems where either photoelectrochemical cells or solid-state photovoltaic apparatuses capture solar energy to drive fuel production are more efficient [20, 29, 30]. This is mainly due to the high efficiency of light-absorbing materials employed. For instance, photovoltaic cells have a solar energy-to-electricity conversion efficiency varying from 16 to 21% in respect of widely used Si panels to greater than 40% in the case of cutting-edge multijunction cells [8, 31]. To date, some of the best artificial photosynthesis systems for fuel production can reach solar energy-to-H_2_ conversion efficiency ranging from ca. 12 to 18% [32–38].

Artificial photosynthesis has been used to drive the reduction of CO_2_ into fuels. Photoelectrochemical reactors developed until now for this purpose produced mostly C1 compounds such as carbon monoxide, methane, methanol, formaldehyde, and formate [39, 40]. One major advantage of biological systems over inorganic apparatuses is their capacity to synthesize a large range of long-chain carbon molecules that can be used as fuels with preferable physicochemical properties or for other chemical applications. Recently, hybrid photosynthesis systems have claimed advantages of both the metabolic versatility of microorganisms and the efficiency of inorganic solar energy capture devices to drive the reduction of CO_2_ into biofuels and other multicarbon compounds [30, 41, 42]. This novel approach is showing promising potentials that could lead to industrial-scale applications in the near future. The purpose of this review is to discuss the recent advances made in the field of hybrid photosynthesis today and to highlight the challenges associated with this technology.

## Principle of hybrid photosynthesis

During hybrid photosynthesis, solar energy is captured by inorganic sunlight absorbers before being used by biological catalysts for driving CO_2_ reduction. Hybrid photosynthesis systems are diverse since they can couple different types of inorganic solar energy capture devices such as solid-state photovoltaics, photoelectrodes, and photocatalyst nanoparticles or several biological catalysts, including autotrophic bacteria or archaea and enzymes.

## Powering MES with solid-state photovoltaic

**Fig. 1:**
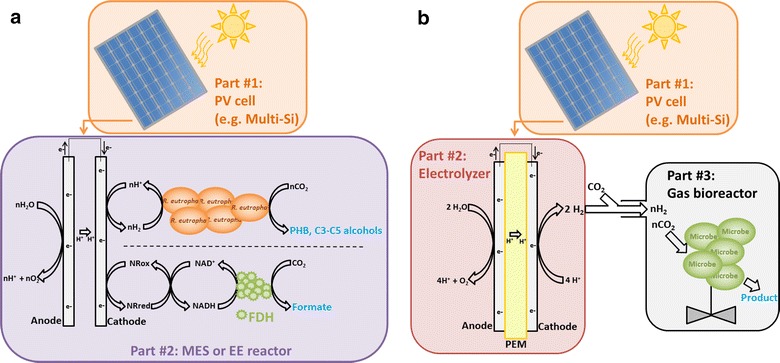
Hybrid photosynthesis with photovoltaic cell. **a** PV-driven MES or EE. In the first MES-based example, *R. eutropha* oxidized H_2_ generated at the cathode to reduce CO_2_ into PHB and/or C3 and C5 alcohols [77]. In the second EE-based example, the reduction of CO_2_ into formate by a formate dehydrogenase via NADH and neutral red (NR) could be powered by a PV cell [108–110, 112]. No membrane was present between the electrodes in the first example, while in the second example, either an ion-exchange membrane (IEM) or salt bridge was present. **b** Alternatively, PV can power H_2_ evolution by an electrolyzer. H_2_ can then be mixed with CO_2_ and fed to a gas bioreactor. Here, a polymer electrolyte membrane (PEM) electrolyzer is depicted

A promising strategy for achieving efficient and productive hybrid photosynthesis process is to power a microbial electrosynthesis (MES) reactor with solid-state photovoltaics (PVs) (Fig. 1a) [29, 43, 44]. The principle behind MES is that autotrophic microbes use reducing equivalents generated by an electrochemical reactor to reduce CO_2_ into biofuels or other chemicals of interest [45, 46]. MES reactors developed until now have different configurations, but the most common one includes an anode and a cathode separated by an ion-exchange membrane and connected by an electric circuit [41]. Protons and electrons are generated by oxidation reactions at the anode, such as water splitting, and the biological oxidation of wastewater or sulfide waste [43, 47–52]. Electrons flow through the electric conduit from the anode to the more negative cathode, while protons migrate through the ion-exchange membrane. Electrons and protons are then acquired by the autotrophic biocatalyst in the cathodic chamber where it reduces CO_2_. MES processes are driven by an external source of electricity that can be generated via renewable energy resources such as wind and solar.

Because of their metabolic versatility, the different biocatalysts employed for MES until now have been shown to produce a large range of products including C1 compounds such as methane and formate as well as multicarbon molecules such as acetate, butyrate, 2-oxobutyrate, propionate, ethanol, 1-butanol, isobutanol, 3-methyl-1-butanol, and wax esters [41, 52–56]. MES biocatalysts are diverse and comprise both pure cultures as well as mixed communities [45]. When mixed communities are employed for MES, acetogens mainly producing acetate by using CO_2_ as electron acceptor via the Wood-Ljungdahl pathway and methanogens generating methane from CO_2_ often become the dominating populations [50, 57–61]. There are many examples of pure culture-driven MES reactors including systems where the biocatalyst was an acetogen, a Fe^2+^-oxidizing bacterium, the ammonia-oxidizer *Nitrosomonas europaea*, the electrogenic bacterium *Geobacter sulfurreducens,* or the bioplastic producer *Ralstonia eutropha* [44, 49, 53, 62–72].

For PV-driven MES, the MES reactor is connected via external wires to an autonomous solid-state PV cell. One of the main reasons why PVs are more efficient at converting solar energy than natural photosynthesis is that they have a larger light absorption range that can stretch from the ultraviolet to the near-infrared [7]. Because of technological innovations and manufacturing scale-up, PV deployment for domestic or industrial usages has increased significantly in the last decade, while costs have decreased concomitantly [73]. In terms of efficiency, market leader multicrystalline Si (multi-Si) PVs can convert 21% of the received solar energy into electricity [8]. Currently, the most efficient PV is a four-junction system (GaInP/GaAs//GaInAsP/GaInAs) still at laboratory scale, which can convert 46% of the received solar energy into electricity [74].

The coulombic efficiency for the production of acetate from CO_2_ by MES is often between 80 and 90% when efficient biocatalysts for MES such as the acetogen *Sporomusa ovata* are employed [43, 63, 65]. Thus, the energetic efficiency of the MES reactor for acetate production could be theoretically around 47% assuming a coulombic efficiency of 85% and a whole electrochemical cell voltage of − 2 V [75]. The theoretical, optimal equilibrium potential in an electrochemical cell for CO_2_ reduction into acetate at the cathode and water splitting at the anode should be − 1.1 V, but because of electrode overpotentials associated with every electrochemical reaction, the practical whole cell voltage should be higher by several hundred millivolts [46, 76]. Thus, powering a MES reactor for acetate production with multi-Si PV or with four-junction PV could result in hybrid photosynthesis systems with solar energy-to-acetate conversion efficiency of ca. 9.9% or ca. 21.6%, respectively (Table 1).

**Table 1:** Examples of hybrid photosynthesis system with microbes as biocatalyst

System	Light harvester	Microbe	Comments	References
PV + MES	Multi-Si PV^a^	*S. ovata*	Graphite electrodes Water splitting Produces acetate and 2-oxobutyrate Efficiency^b^: 8.5–9.9%	[43]
PV + MES	Multi-Si PV	*R. eutropha*	Co-Pi anode/Co-P cathode Water splitting PHB efficiency: 7.6–8.9% C3–C5 alcohols efficiency: 7.1–8.3%	[77]
PBEC	Si nanowire photocathode TiO_2_ photoanode	*S. ovata*	Water splitting 1.2 g L^−1^ day^−1^ acetate Efficiency: 0.38% PHB, *n*-butanol and isoprenoids were produced from acetate in a 2nd reactor	[98]
PBEC	*n* ^+^/*p*-Si photocathode	*M. barkeri*	Pt gauze anode Water splitting 5.86 mL day^−1^ CH_4_	[90]
PBEC	*p*-InP photocathode *n*-TiO_2_ nanowire/FTO photoanode	*M. barkeri*	Water splitting 0.58 mL day^−1^ CH_4_	[90]
PC	CdS nanoparticles	*M. thermoacetica*	Cysteine as e^−^ donor 0.48 mM day^−1^ acetate	[101]
PC	CdS nanoparticles TiO_2_ nanoparticles	*M. thermoacetica*	Z-scheme Water splitting Cysteine as e^−^ shuttle Cocatalyst MnPc 1.2 mM day^−1^ acetate	[102]

*PV* photovoltaic, *MES* microbial electrosynthesis, *PBEC* photobioelectrochemical cell, *PC* photocatalyst-driven system

^a^Coupling of the PV with the MES system has not been tested experimentally. Solar energy-to-electricity conversion efficiency of multi-Si PV was assumed to be 18–21%

^b^Efficiency refers to solar energy-to-product conversion efficiency

Recently, Liu et al. developed a MES system for the production of polyhydroxybutyrate (PHB) and C3–C5 alcohols from CO_2_ with a Co-Pi anode, a Co–P alloy cathode, and *Ralstonia eutropha* as the microbial catalyst (Fig. 1a) [77]. This electrode tandem was employed because it significantly lessens the overpotential required for water splitting and it is also nontoxic for bacteria. *R. eutropha* was used as the biocatalyst because it can produce significant quantity of PHB from CO_2_ and it can also be genetically engineered [78, 79]. Interestingly, relatively good energy efficiencies of 42% for PHB, 39% for isopropanol, and 27% for C4 and C5 alcohols were achieved with this MES system. Based on these results, the authors calculated that powering their MES reactor with a PV device that has a solar energy-to-electricity conversion efficiency of 18% would result in a hybrid photosynthesis system with a solar energy-to-chemicals conversion efficiency of 7.6% for PHB and 7.1% for C3–C5 alcohols.

One advantage of MES in the context of hybrid photosynthesis is that physical contact between the microbial catalyst and the cathode may reduce the electrical energy and electrode overpotentials required for the generation of reducing equivalents at the cathode by the electrochemical reactor, making the whole system more efficient. For instance, it has been shown that the presence of microbes in the cathodic chamber of a MES reactor can accelerate the evolution of H_2_, which is often the main electron shuttle in this type of system [80, 81]. Furthermore, it has been suggested that electrons can be transferred directly from the cathode to microbes without the need for electron shuttles, which is an electron-transfer mechanism that would also require less electrical energy [43, 68, 71].

## Coupling photovoltaic with an electrolyzer to produce H_2_ or CO for gas fermentation

As an alternative to MES, it may be advantageous in terms of efficiency and productivity to assemble a hybrid photosynthesis system, where solid-state PVs are powering an electrolyzer splitting water to provide H_2_ to a gas fermentation reactor for the reduction of CO_2_ by an autotrophic biocatalyst (Fig. 1b) [41]. Nowadays, scale-up commercial electrolyzers using proton-exchange membrane (PEM) have energetic efficiencies between 65 and 70% [82]. This means that if an acetogen or another microbial catalyst converts ca. 80–90% of electrons from H_2_ into acetate or other products, the solar energy-to-specific product conversion efficiency of a system combining multi-Si PVs, a PEM electrolyzer, and a H_2_:CO_2_ gas fermentation reactor could be 11–13%. Compared with a PV-MES approach, the main advantage of this system is that the specialized electrolyzer can maintain a high flux of reducing equivalents toward the gas bioreactor, preventing productivity bottleneck associated with MES [41].

Electrolyzers powered by PVs could also be used to reduce CO_2_ into CO, which can then be fed to a gas fermentation reactor for the production of biofuels by acetogens [82]. This approach is particularly advantageous since CO-rich gas feeds are required by acetogen-mediated gas fermentation reported until now to achieve the highest ethanol production yields [83]. Reported CO_2_-to-CO reduction efficiencies for CO_2_ electrolyzers being developed today are greater than 80% [84, 85]. Besides CO, CO_2_ electrolyzers are being developed for the production of other C1 molecules, such as formate or methanol, which could also be used by microbial catalysts as electrons and carbon sources for the synthesis of multicarbon molecules including C2 and above biofuels [86–89].

## Photobioelectrochemical cells with microbes

**Fig. 2:**
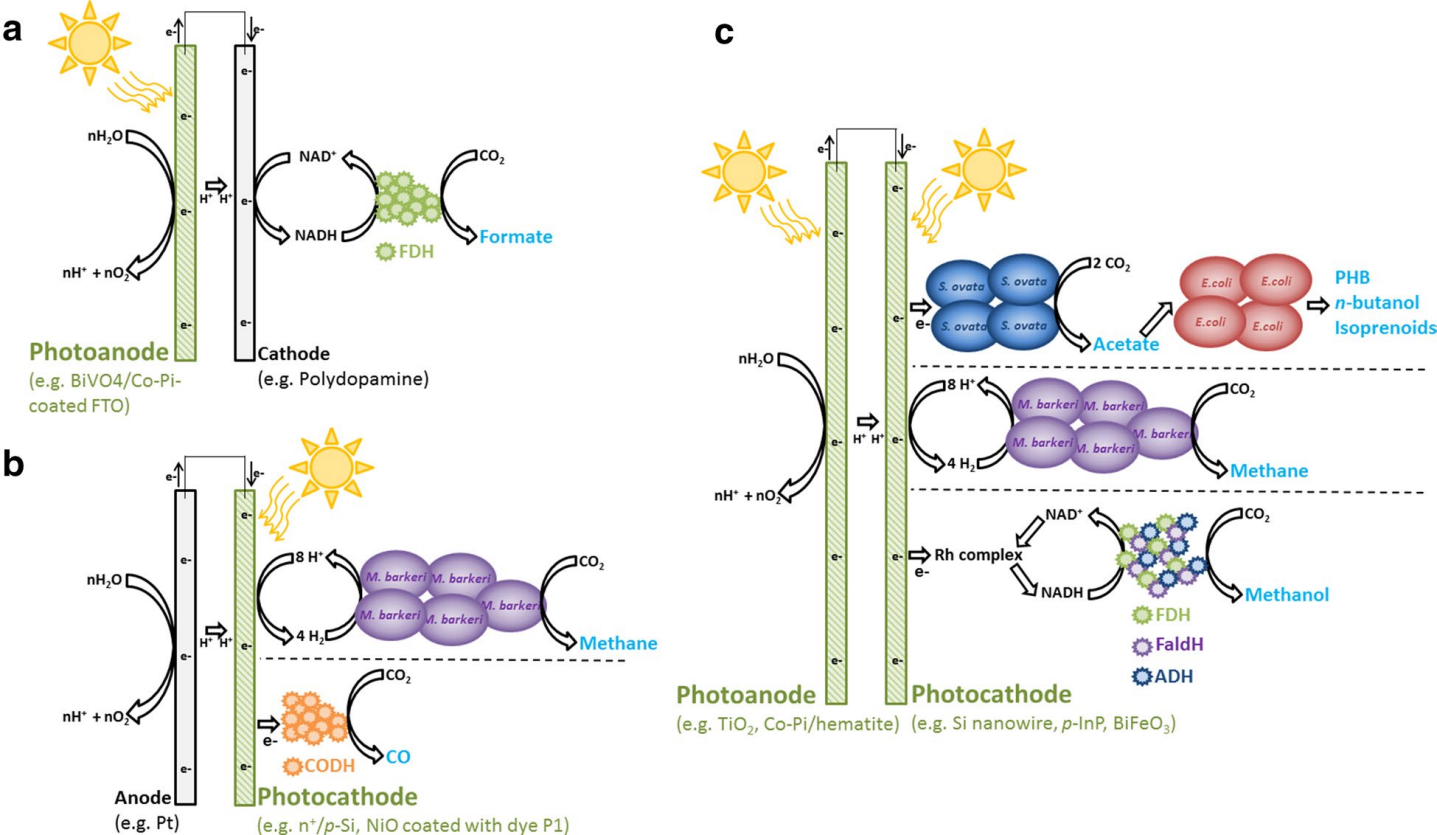
Photobioelectrochemical cells (PBECs). **a** PBEC with a photoanode. CO_2_ is reduced into formate by a formate dehydrogenase (FDH) via NADH [114]. In this system, an IEM separated the electrodes. **b** PBEC with a photocathode. In the first example, *M. barkeri* oxidized H_2_ coming from the photocathode to reduce CO_2_ into methane [90]. In the second example, a carbon monoxide dehydrogenase (CODH) acquired electrons directly from the photocathode to reduce CO_2_ into CO [113]. An IEM was present in example 1 while example 2 was a single-chamber reactor. **c** PBEC with a photoelectrodes tandem. In the first example, *S. ovata* acquired electrons directly from a photocathode to reduce CO_2_ into acetate [98]. Acetate is then converted to other products by *E. coli*. In the second example, *M. barkeri* reduced CO_2_ into methane with H_2_ from the PEC [90]. In the third example, methanol is produced from CO_2_ by an enzymatic cascade acquiring electrons from a photocathode via a rhodium complex and NADH [115]. Examples 1 and 2 comprised an IEM, while photoelectrodes in example 3 were separated by a salt bridge

Multiple hybrid photosynthesis reactors developed in the recent years can be classified as photobioelectrochemical cells (PBECs). In these systems, light absorbers immersed in the electrolytes capture solar energy that will be used to split water at the (photo)anode and to generate reducing equivalents at the (photo)cathode (Fig. 2) [90]. Microbial or enzymatic catalysts will then reduce CO_2_ by acquiring reducing equivalents from the (photo)cathode. PBECs are derived from photoelectrochemical cells (PEC), which have been extensively investigated over the last 50 years [91]. These systems do not include a biocatalyst and have been mainly developed for the production of H_2_ to store energy from sunlight [92, 93]. PEC and PBEC can include a photoanode, a photocathode, or both in a tandem configuration (Fig. 2a–c) [94]. Photoanodes are made of *n*-type semiconducting materials accumulating photoexcited holes that are used for water oxidation. The surface of the photoanode is often modified with oxygen evolution catalysts such as IrO_2_ to accelerate water splitting and improve photocurrent in the system. Electrons from oxidation reactions at the anode are then transferred via an electric circuit to the cathode. If a photocathode is included in the PEC or PBEC, it is made of a *p*-type semiconducting material where electrons coming from the (photo)anode are photoexcited to higher energy level. For H_2_ evolution, the photocathode is often modified with a hydrogen evolution catalyst such as platinum or earth-abundant and nonprecious metals [35, 95–97].

To our knowledge, the first functional microbial PBEC reported in the literature in 2015 was developed by Liu and coworkers at the University of California, Berkeley to produce *n*-butanol, PHB, or isoprenoids from CO_2_ and solar energy (Table 1) [98]. This hybrid photosynthesis system consisted of light-absorbing Si nanowire photocathode and TiO_2_ photoanode separated by a cation-exchange membrane (Fig. 2c). Both the photocathode and the photoanode were exposed to light via quartz windows. The surface of the Si nanowire photocathode had three modifications: (1) a doped n^+^ layer to improve photovoltage, (2) a TiO_2_ layer to increase stability at neutral pH, and (3) a Ni and Pt layer to accelerate the transfer of reducing equivalents to the biocatalyst. The acetogen *Sporomusa ovata* was selected as the microbial catalyst because, as mentioned previously, it is particularly efficient at reducing CO_2_ into acetate with the cathode of an MES reactor as the sole electron source [43, 65, 66, 99]. Interestingly, *S. ovata* is a strict anaerobe, but it was still able to synthesize acetate in this PBEC even though the 10% CO_2_ feed also contained 21% O_2_. According to the authors, this occurrence is due to the conformation of the Si nanowire array photocathode, which harbors local anaerobic conditions. Under simulated sunlight, the PBEC was stable for more than 120 h with a photocurrent of 0.3 mA cm^−2^. Acetate titer reached greater than 6 g/L, and the solar energy-to-acetate efficiency of this proof-of-concept reactor was 0.38%. Acetate produced by the PBEC was then used as feedstock in a downstream bioprocess by recombinant *E. coli* for the production of more valuable chemicals with longer carbon chains.

The same author group also developed two PBECs driven by the methanogen *Methanosarcina barkeri* for the production of methane from CO_2_ and solar energy (Table 1) [90]. In the first PBEC, only a portion of the electrical energy necessary for water splitting was derived from sunlight. The anode of platinum gauze was combined with a *n* ^+^/*p*-Si photocathode coated with a nickel-molybdene alloy evolving H_2_ to drive *M. barkeri* metabolism (Fig. 2b). This system produced 17.6 mL CH_4_ over a period of 3 days and demonstrated that photoexciting electrons at a photocathode reduced the overpotential required for chemicals production using a bioelectrochemical reactor. The second methane-producing PBEC was powered exclusively by sunlight. It comprised a *n*-TiO_2_ nanowire/fluorine-doped tin oxide (FTO) photoanode separated by an anion-exchange membrane from a *p*-InP photocathode coated with platinum for H_2_ evolution and colonized by *M. barkeri* (Fig. 2c). In this experiment, a full-light spectrum illuminated the photoanode before reaching the photocathode. Because of the sensitivity of methanogens to blue light, the PBEC reactor was also modified using a light filter inserted between both photoelectrodes. The sunlight-driven PBEC produced 1.75 mL CH_4_ in 3 days.

PBEC using living cells as biocatalysts are still at an early stage of development as illustrated by the low productivity and solar energy-to-chemical conversion efficiency observed with these systems. Significant challenges must be overcome to reach the full potential of microbial PBEC including the development of biocompatible photocathodes that are optimal for both sunlight capture and electrons transfer to the biocatalyst. Based on the results in the literature until now, it could be argued that hybrid photosynthesis may be better served by a more compartmentalized system such as PV-driven MES or PV coupled with an electrolyzer and a gas bioreactor. With these approaches, components responsible for the conversions of solar energy-to-electrical and electrical-to-chemical energy can be optimized without consideration for maintaining conditions favorable to living cells that may limit performance and efficiency. Likewise, bioreactor components catalyzing the conversion of electrical or chemical energy into target molecules could be improved without consideration for sunlight capture. Still, PBEC like other PEC-based technologies is being pursued by the research community because, after considerable improvement, it may become more cost effective than PV-based technologies [100].

## Coupling inorganic photocatalysts with living cells

**Fig. 3:**
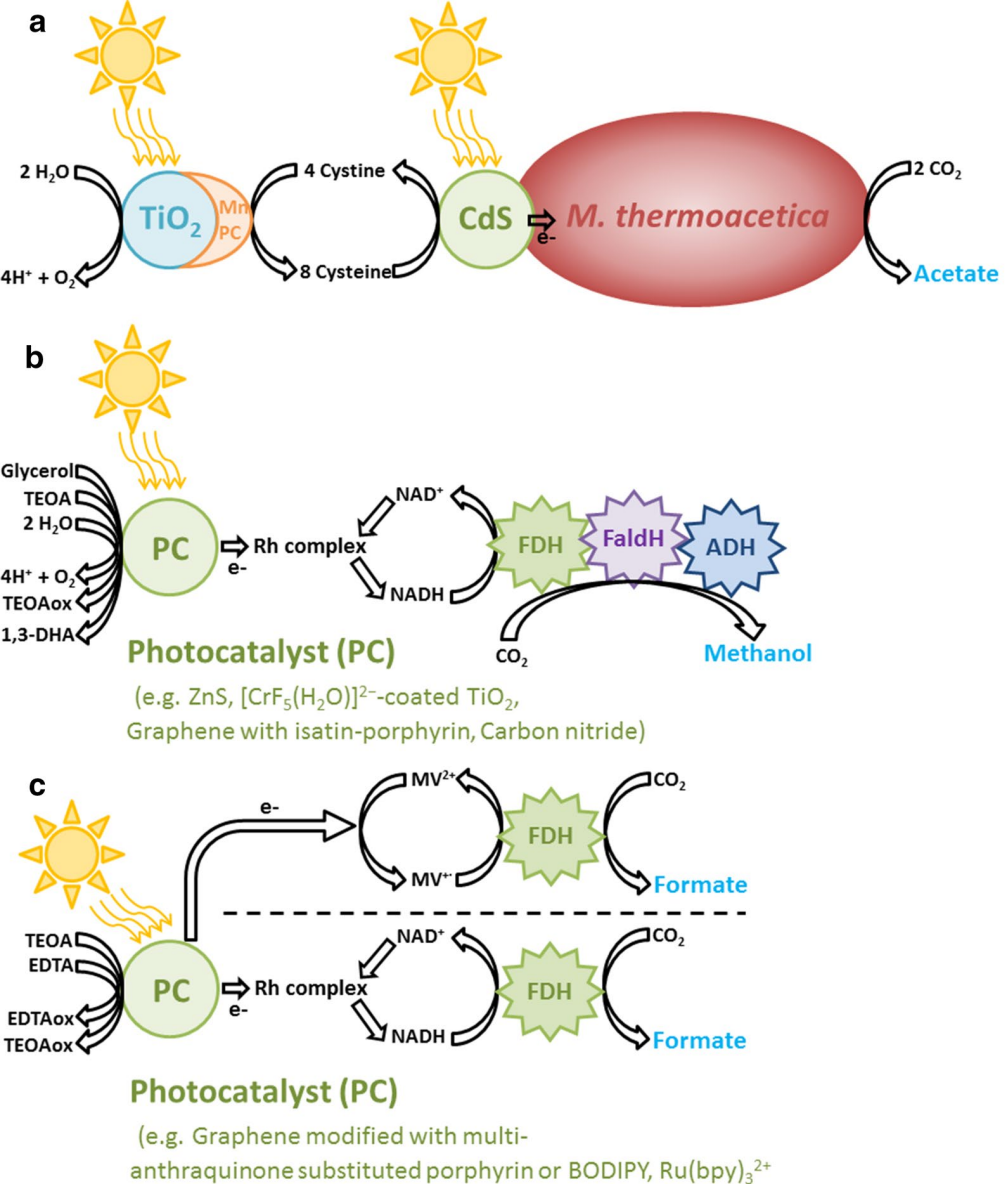
Hybrid photosynthesis using photocatalyst. **a** Photocatalyst-driven microbial CO_2_ reduction. In a tandem system, water is oxidized by a TiO_2_ photocatalyst [102]. Electrons are transferred from TiO_2_ to cystine via a MnPC cocatalyst. The resulting cysteine is then oxidized by a CdS photocatalyst. *M. thermoacetica* reduced CO_2_ into acetate with electrons from CdS. **b** Photocatalyst-driven enzymatic reduction of CO_2_ to methanol [116–119]. **c** Photocatalyst-driven enzymatic reduction of CO_2_ to formate. In the first example, electrons are transferred from the photocatalyst to the formate dehydrogenase (FDH) via methylviologen [123]. In the second example, electrons are transferred from the photocatalyst to FDH via a rhodium complex and NADH [120, 121]

Recently, two hybrid photosynthesis systems comprising photocatalyst nanoparticles driving the microbial reduction of CO_2_ into multicarbon compounds were developed by Sakimoto and coworkers (Table 1). In both systems, the microbial catalyst employed was the thermophilic *Moorella thermoacetica*, an acetogen mainly reducing CO_2_ into acetate also capable of acquiring electrons from a cathode [53, 101, 102]. The energy required to drive the autotrophic metabolism of *M. thermoacetica* came from light-harvesting CdS nanoparticles, which can oxidize the redox mediator cysteine. CdS nanoparticles were precipitated by *M. thermoacetica* and could be observed in clusters at the surface of microbial cells. The first system developed according to this strategy produced ca. 1.2 mM acetic acid in 2.5 days under low-intensity-simulated sunlight. In the second system, a tandem “Z-scheme” architecture was adopted where cystine resulting from the oxidation of cysteine was reduced by a water-splitting catalyst composed of manganese(II) phthalocyanine (MnPC) cocatalyst attached to light-harvesting TiO_2_ nanoparticles (Fig. 3a). This system could produce ca. 0.6 mM acetic acid within half a day of illumination. Compared with PV-based approaches and PBEC, coupling photocatalyst nanoparticles with microbial catalysts does not appear to be very productive. However, this technology is still in its infancy, and after significant improvement in terms of productivity and efficiency, it could become more cost effective than competing approaches since it requires only a single bioreactor without electrodes, PV cells, or electrolyzers.

## Hybrid photosynthesis with enzymes

Besides living cells, hybrid photosynthesis systems for CO_2_ reduction could also be assembled with purified enzymes as biocatalyst (Table 2). This includes PV-based approaches, enzyme-driven PBEC, as well as strategies coupling inorganic photocatalysts with enzymes. Until now, enzyme-driven hybrid photosynthesis has only been developed for the reduction of CO_2_ into small C1 molecules including CO, formate, and methanol [103]. With the exception of methanol, these molecules cannot be used directly as fuel, but can serve as more reduced substrates than CO_2_ for downstream fuel-production processes. The narrow range of products is one of the disadvantages of enzyme-driven hybrid photosynthesis, which is also stymied by the cost and difficulties associated with enzyme purification and short lifetime [104, 105]. However, compared with living cell-driven hybrid photosynthesis, enzyme-based systems have also several advantages: (1) there is no requirement for complex microbial growth medium containing diverse nutrients augmenting cost, (2) products are more specific since living cells harbor multiple metabolic pathways leading to the synthesis of unwanted byproducts, and (3) energetic efficiency is not decreased by the need to generate and maintain biomass.

**Table 2:** Examples of hybrid photosynthesis system with enzymes as biocatalyst

System	Light harvester	Enzyme	Comments	References
PBEC	NiO photocathode with dye P1	CODH	Pt wire anode Water splitting Produces carbon monoxide	[113]
PBEC	BiVO_4_- and Co-Pi-coated FTO photoanode	FDH	Polydopamine cathode Water splitting NADH as cofactor Produces formate Efficiency^a^: 0.042%	[114]
PBEC	BiFeO_3_ photocathode Co-Pi/hematite photoanode	FDH/FaldH/ADH	Z-scheme Water splitting Rhodium complex used as redox mediator Cofactor NADH 220 μM h^−1^ methanol	[115]
PC	ZnS nanorods	FDH/FaldH/ADH	Glycerol as e^−^ donor Cofactor NADH Produces methanol	[116]
PC	TiO_2_ nanoparticles coated with [CrF_5_(H_2_O)]^2−^	FDH/FaldH/ADH	Glycerol or water as e^−^ donor Rhodium complex used as redox mediator Cofactor NADH Produces methanol	[117]
PC	Graphene modified with isatin–porphyrin chromophore	FDH/FaldH/ADH	TEOA as e^−^ donor Rhodium complex used as redox mediator Cofactor NADH 7.47 μM h^−1^ methanol	[118]
PC	Carbon nitride array	FDH/FaldH/ADH	TEOA as e^−^ donor Rhodium complex used as redox mediator Cofactor NADH 45 nM h^−1^ methanol	[119]
PC	Graphene modified with multi-anthraquinone substituted porphyrin	FDH	TEOA as e^−^ donor Rhodium complex used as redox mediator Cofactor NADH 55.5 μmol h^−1^ formate	[120]
PC	Graphene modified with the BODIPY molecule	FDH	TEOA as e^−^ donor Rhodium complex used as redox mediator Cofactor NADH 72 μmol h^−1^ formate	[121]
PC	Photosensitizer Ru(bpy)_3_^2+^	FDH	EDTA as e^−^ donor Methylviologen used as redox mediator Immobilization inside porous glass improved performance 15 mM h^−1^ formate Efficiency: 0.22%	[123]

*PBEC* photobioelectrochemical cell, *CODH* carbon monoxide dehydrogenase, *FDH* formate dehydrogenase, *FaldH* formaldehyde dehydrogenase, *ADH*: alcohol dehydrogenase, *PC* photocatalyst-driven system, *TEOA* triethanolamine, *EDTA*: ethylenediaminetetraacetic acid

^a^Efficiency refers to solar energy-to-product conversion efficiency

## Powering enzymatic electrosynthesis from CO_2_ with photovoltaics

PVs could be used to power the enzymatic reduction of CO_2_ at the cathode of bioelectrochemical reactors. Enzymatic electrosynthesis (EE) is very similar to MES with the exception that the biocatalyst is an enzyme instead of a microbe (Fig. 1a) [106]. Until now, only formate and methanol have been produced from CO_2_ by EE. In the first report on EE, the tungsten-containing formate dehydrogenases of *Syntrophobacter fumaroxidans* appeared to accept electron directly from a cathode for the reversible reduction of CO_2_ into formate [107]. In systems developed later, the redox mediator, neutral red, transferred electrons from a carbonaceous cathode for the regeneration of NADH, which was then oxidized by a formate dehydrogenase [108–110]. Alternatively, a rhodium complex [CpRh(bpy)(H_2_O)]^2+^ was employed to transfer electrons from a copper electrode to the cofactor NADH [111]. The neutral red-based EE system developed by Addo and coworkers included a formaldehyde dehydrogenase and an alcohol dehydrogenase with the formate dehydrogenase to establish an enzymatic cascade enabling the production of methanol from CO_2_ [109]. This system also comprised a carbonic anhydrase for the conversion of dissolved CO_2_ into bicarbonate, which accelerated the EE reaction.

Most formate-producing EE processes described in the literature had a coulombic efficiency below 13% with the exception of a system developed by Zhang and coworkers. They immobilized the formate dehydrogenase of *Candida boidinii* with Nafion micelles at the surface of a cathode coated with neutral red and poised at − 0.8 V (vs. SHE). Nafion micelles increased the lifetime of the fragile formate dehydrogenase and protected its enzyme activity. The reported coulombic efficiency of this system was 77.08% with a production rate of 64.71 mg L^−1^ h^−1^ over a period of 2 h [112]. Within this range of coulombic efficiency, powering EE with PV could result in a hybrid photosynthesis system with a significant solar energy-to-formate conversion efficiency.

## Photobioelectrochemical cells with enzymes

Examples of PBEC driven by enzyme include a system where the carbon monoxide dehydrogenase I of the thermophilic and chemolithotrophic bacterium *Carboxydothermus hydrogenoformans* can reduce CO_2_ into CO with electrons coming from a photocathode (Fig. 2b, Table 2) [113]. This electrode made of a *p*-type semiconductor NiO was photosensitized using the organic dye P1 responsive to visible light. In this PBEC, photoexcited electrons from the photocathode were first transferred to the FeS clusters of the carbon monoxide dehydrogenase I before reaching the Ni4Fe-4S active site of the enzyme. The counter-electrode, oxidizing water in this system, was made of platinum wire. In a second example, Lee and coworkers designed a PBEC for the reduction of CO_2_ into formate, coupling a photoanode splitting water by means of a cathode made of polydopamine coated with the formate dehydrogenase of *C. boidini* and its cofactor NADH (Fig. 2a) [114]. Polydopamine was chosen as cathode material because it is biocompatible, and it has a good charge-transfer capacity. Electrons from the cathode were transferred to the active site of the formate dehydrogenase via the reduction of NAD^+^ into NADH. The photoanode was a FTO electrode coated with the visible-light-absorber BiVO_4_ and with the water oxidation catalyst Co-Pi. This system had a solar energy-to-formate conversion efficiency of 0.042% and was stable for at least 24 h.

Recently, Kuk and coworkers designed an enzyme-driven PBEC for the reduction of CO_2_ to methanol with a visible light-absorbing photocathode and photoanode tandem (Fig. 2c; Table 2) [115]. The photocathode was made of a *p*-type perovskite semiconductor BiFeO_3_, and the photoanode comprised hematite and Co-Pi. Photoexcited electrons from the photocathode regenerated the cofactor NADH via a rhodium complex as redox mediator. Water was oxidized at the photoanode by the Co-Pi catalyst. With this system, a high visible light-driven methanol conversion output of 220 μM h^−1^ was observed, but only when applying an electrical bias of 0.8 V.

## Coupling inorganic photocatalysts with enzymes

The three enzymes cascade used to produce methanol have also been coupled with different photocatalytic nanoparticles driving the photochemical regeneration of NADH (Table 2; Fig. 3b). For instance, Dibenedetto and coworkers proposed a system coupling methanol production with NAD^+^ reduction by a ZnS photocatalyst using glycerol as electron donor and illuminated by light at the border of the visible and UV spectra [116]. The same group later assembled a system relying on a visible-light-absorbing photocatalyst made of TiO_2_ coated with the photosensitizer [CrF_5_(H_2_O)]^2−^ for the regeneration of NADH followed by methanol production [117]. With this system, water could be used as the electron donor for NAD^+^ reduction, but the photocatalyst was more efficient with glycerol. Furthermore, adding a rhodium complex to serve as redox mediator significantly improved NADH regeneration. Other visible-light-harvesting photocatalysts developed for NADH regeneration coupled with the enzymatic production of methanol include graphene modified with isatin–porphyrin chromophore and carbon nitride semiconductor array combined with a rhodium complex [118, 119]. These two systems used triethanolamine (TEOA) as electron donor for the photoregeneration of NADH. The graphene modified with isatin–porphyrin photocatalyst was the most productive system with 11.21 μM methanol being produced from CO_2_ within 90 min.

Photocatalytic particles have been used likewise to regenerate NADH for the enzymatic production of formate (Fig. 3c; Table 2). For this purpose, Yadav and coworkers developed visible-light-absorbing photocatalysts coupling the oxidation of TEOA with NADH regeneration via a rhodium complex as redox mediator. These photocatalysts were made of graphene modified with either multianthraquinone-substituted porphyrin or the light-harvesting BODIPY compound [120, 121]. Within 2 h of operation, the two formate dehydrogenase-coupled photocatalysts could produce 111 and 144 μmol of formate, respectively. Graphene was used in the fabrication of photocatalyst composites such as the three examples described here, because it has a high charge-transport efficiency and an excellent specific surface area, which are both beneficial for photocatalytic and photovoltaic systems [122].

Recently, Noji and coworkers developed a light-induced formate production system where the redox mediator methylviologen transferred electrons from the photosensitizer Ru(bpy)_3_^2+^ to a formate dehydrogenase reducing CO_2_ (Fig. 3c) [123]. In this case, the photosensitizer oxidized EDTA for the reduction of methylviologen. The formate production rate and the solar energy-to-formate conversion efficiency of this system were 0.18 mM h^−1^ and 0.016%, respectively. Interestingly, when the photosensitizer, methylviologen, and the formate dehydrogenase were immobilized inside the nanopore of porous glass plates, the three components became denser, the production rate was increased to 15 mM h^−1^, and the efficiency was also significantly improved to 0.22%. Beside low productivity and efficiency, the usage of electron donors other than water for the biophotocatalytic production of methanol or formate via enzymes is one of the major hurdles between this technology and practical applications, especially since compounds such as EDTA and TEOA need to be regenerated after oxidation.

## Conclusions

Hybrid photosynthesis is a novel technology still at an early stage of development, regardless of whether it can be combined with more mature technologies like PV cell and electrolyzer. In our opinion, it is premature to make economic comparison between the various types of hybrid photosynthesis systems described here since multiple technical parameters could be dramatically improved in the near future. Increasing the efficiency and productivity of hybrid photosynthesis systems need to be done in part via the optimization of the light-harvesting inorganic apparatus and of the biocatalyst. To reach this objective, cheaper and better semiconductor materials must be developed for the fabrication of biocompatible photocatalysts or photoelectrodes, and novel biocatalysts accelerating the light-driven reduction of CO_2_ into specific products must be engineered through well-thought synthetic biology approaches. In addition, to reach the practical voltage required for water oxidation and microbial CO_2_ reduction, it may be advantageous to design novel reactor architecture combining several of the hybrid photosynthesis strategies described here. For instance, coupling the photocathode of a PBEC and its associated biocatalyst with a PV cell could lead to higher energetic efficiency [32, 35]. Another promising avenue of research for the transformation of solar energy into multicarbon biofuels that should be investigated is the conversion of solar heat into reducing equivalents, which could then be used to drive the biological reduction of CO_2_ [124]. Since a large fraction of solar energy is converted into heat, a hybrid photosynthesis system harvesting both sunlight and solar heat could lead to a significant increase in productivity and in solar energy-to-biofuels conversion efficiency.

Development of robust, safe, cost-effective, productive, and efficient hybrid photosynthesis systems could be a major technological breakthrough. However, many important challenges other than improving efficiency and performance remained before possible scaling up. This includes limitation associated with the usage of atmospheric CO_2_ as carbon source [8]. CO_2_ is present in low concentration in the atmosphere and would probably have to be enriched, which could significantly increase cost associated with hybrid photosynthesis. A more reasonable solution would be to use CO_2_-rich flue gas as feedstock, which can be obtained directly from industrial emitters such as ceramic, glass, steel, and power plant, or from anaerobic digestion plant. A second issue that could prevent hybrid photosynthesis from becoming economically feasible is product separation and extraction from the electrolyte/growth medium, which can account for the major fraction of the cost associated with microbial bioproduction plants [125]. A potential cheaper solution that has been implemented successfully in bioelectrochemical reactors for carboxylic acids would be to separate products in PBEC or in PV-based reactor using integrated membrane electrolysis [126, 127]. Based on these observations, it is clear that research on hybrid photosynthesis has many questions left unanswered and obstacles to overcome. Nevertheless, this field of activity is under rapid development and is showing exciting promises for the future of bioenergy.

## Abbreviations

ADH: alcohol dehydrogenase
CODH: carbon monoxide dehydrogenase
EDTA: ethylenediaminetetraacetic acid
EE: enzymatic electrosynthesis
FaldH: formaldehyde dehydrogenase
FDH: formate dehydrogenase
FTO: fluorine-doped tin oxide
IEA: International Energy Agency
IEM: ion-exchange membrane
MES: microbial electrosynthesis
MnPC: manganese(II) phthalocyanine
NR: neutral red
PBEC: photobioelectrochemical cell
PEC: photoelectrochemical cell
PHB: polyhydroxybutyrate
PEM: polymer electrolyte membrane
PV: photovoltaic
SHE: standard hydrogen electrode
TEOA: triethanolamine
